# Cytocompatibility and Antibacterial Properties of Capping Materials

**DOI:** 10.1155/2014/181945

**Published:** 2014-05-18

**Authors:** Claudio Poggio, Carla Renata Arciola, Riccardo Beltrami, Annachiara Monaco, Alberto Dagna, Marco Lombardini, Livia Visai

**Affiliations:** ^1^Department of Clinical-Surgical, Diagnostic and Pediatric Sciences, University of Pavia, Policlinico “San Matteo”, Piazzale Golgi 3, 27100 Pavia, Italy; ^2^Research Unit on Implant Infections, Rizzoli Orthopedic Institute, DIMES of the University of Bologna Via di Barbiano 1/10, 40136 Bologna, Italy; ^3^Department of Brain and Behavioral Sciences, University of Pavia, Via Bassi 21, 27100 Pavia, Italy; ^4^Department of Molecular Medicine, Center for Tissue Engineering (CIT), INSTM UdR of Pavia, University of Pavia, Viale Taramelli 3/b, 27100 Pavia, Italy; ^5^Department of Occupational Medicine, Ergonomy and Disability, Laboratory of Nanotechnology, Salvatore Maugeri Foundation, IRCCS, Via S. Boezio 28, 27100 Pavia, Italy

## Abstract

The aim of this study was to evaluate and compare the antimicrobial activity and cytocompatibility of six different pulp-capping materials: Dycal (Dentsply), Calcicur (Voco), Calcimol LC (Voco), TheraCal LC (Bisco), MTA Angelus (Angelus), and Biodentine (Septodont). To evaluate antimicrobial activity, materials were challenged *in vitro* with *Streptococcus mutans*, *Streptococcus salivarius*, and *Streptococcus sanguis* in the agar disc diffusion test. Cytocompatibility of the assayed materials towards rat MDPC-23 cells was evaluated at different times by both MTT and apoptosis assays. Results significantly differed among the different materials tested. Both bacterial growth inhibition halos and cytocompatibility performances were significantly different among materials with different composition. MTA-based products showed lower cytotoxicity and valuable antibacterial activity, different from calcium hydroxide-based materials, which exhibited not only higher antibacterial activity but also higher cytotoxicity.

## 1. Introduction

Direct pulp-capping is a procedure for covering the exposed surface of the pulp to maintain its vitality and preserve its functional and biologic activities. The ultimate goal of capping material has been widely recognized as inducing pulp cells to form hard tissue [1].

Several materials such as calcium hydroxide-based materials and, more recently, mineral trioxide aggregate (MTA) are commonly recommended to seal communications between the exposed pulp and the oral cavity [2–6].

Calcium hydroxide-based materials are the most popular agents for direct and indirect pulp-capping, given their ability to release hydroxyl (OH) and calcium (Ca) ions upon dissolution [7–9]. It is assumed that they lead to initial change that causes undifferentiated cells within the pulp to differentiate into odontoblasts, which then form a hard tissue barrier at the pulp exposure site [10, 11]. The formation of reparative dentine in response to calcium hydroxide may not be due to the bioinductive capacity of the material, but it is due to the result of a defense mechanism by the pulp induced by the irritant nature of calcium hydroxide [12–15].

Dycal (Dentsply, Milford, DE, USA) is a self-setting (2.5-3.5 min) radiopaque calcium hydroxide-based material employed in direct and indirect pulp-capping procedures. Its alkaline pH (pH 9–11) stimulates the formation of secondary dentine when the material is in direct contact with the pulp. Its toxicity to pulp cells is well documented [16].

Mineral trioxide aggregate (MTA) cement has a composition similar to that of Portland cement (PC). Both are composed of calcium phosphate, calcium, and silicon oxide. MTA, in addition, contains bismuth oxide, which provides radiopacity. MTA is a powder that contains trioxides and hydrophilic particles, which set in the presence of moisture [17].

MTA was first introduced as a water-based grey-colored root-end filling and perforation repair material [18]. It has high pH but low compressive strength; depending on its powder/liquid ratio, MTA possesses some antibacterial properties [19]. MTA seals well and is a biocompatible cement; hydroxyapatite crystals form over MTA in contact with tissue fluid [17, 20]. However, it has some known drawbacks, such as a long setting time, high price, and potential discoloration [21]. Moreover, MTA cements exhibit calcified tissue-conductive activity and facilitate the differentiation of human orofacial mesenchymal stem cells [22] and the mineralization process in human dental pulp cells. They also have the potential to be used as pulp-capping materials.

MTA-Angelus (Angelus, Londrina, PR, Brazil) is bioactive, biocompatible, and self-setting hydrophilic calcium silicate cement [17, 19, 22] now successfully used for direct pulp-capping [21, 23]. It contains type III Portland cement, bismuth oxide, tricalcium silicate, dicalcium silicate, and tricalcium aluminate tetracalcium aluminoferrite. MTA is more effective and better than calcium hydroxide materials, as it has an enhanced interaction with dental pulp tissue [24] with limited pulp tissue necrosis (less caustic effect) shortly after its application and less pulp inflammation [25]. MTA facilitated the proliferation/differentiation of human dental pulp cells [24] and exhibited calcified tissue-conductive activity with the ability to stimulate faster complete dentine bridge formation and new hard tissue formation, thus revealing a material endowed with the potential for integration into the peri-implant tissues and preventing infection [25–28].

TheraCal (Bisco Inc., Schaumburg, IL, USA) is a new light-cured, resin-modified, calcium silicate-filled base/liner material designed with direct and indirect pulp-capping. It contains polymerizable methacrylate monomers, Portland cement type III, polyethylene glycol dimethacrylate, and barium zirconate. TheraCal is well tolerated by immortalized odontoblast cells [29].

Calcicur (Voco GmbH, Cuxhaven, Germany) is a ready-to-use radiopaque water-based calcium hydroxide paste. It contains 45% calcium hydroxide and exhibits a high alkalizing pH correlating positively with the Ca(OH)_2_ mass fraction contained in it.

Calcimol LC (Voco GmbH, Cuxhaven, Germany) is a light-cured, resin-modified calcium ion releasing base liner and pulp-capping material. It contains urethane dimethacrylate resin, calcium dihydroxide, dimethylaminoethyl-methacrylate, and triethyleneglycol dimethacrylate (TEGDMA).

Biodentine (Septodont, Saint-Maur-des-Fosses, France) is a bicomponent material. The powder contains tricalcium silicate, calcium carbonate, and zirconium oxide; the liquid contains water, calcium chloride (accelerator), and modified polycarboxylate. Biodentine is an interesting alternative to conventional calcium hydroxide-based materials. It offers advantages for direct pulp-capping and, in properly selected cases, may contribute to the long-term maintenance of tooth vitality [30].

Among the properties that an endodontic sealer or a material for pulp-capping should have, the antibacterial activity can influence the success of the treatment. Furthermore, the materials that possess both optimum flow ability and antibacterial properties might theoretically eliminate residual microorganisms located around exposed pulp without damaging pulp tissue.

One of the objectives of operative dentistry is at the same time to maintain the pulp health in compromised teeth, thus reducing the need for root canal treatment and the potential for unwanted* sequelae* such as tooth loss. As reported above, methods used for this purpose are direct pulp-capping and pulpotomy, which consist of placement of biocompatible materials and bioinductors on the exposed pulp tissue to preserve its health and stimulate repair by mineralized tissue formation [2, 31]. A fundamental feature for these materials is biocompatibility, which includes antibacterial and healing induction properties, cytocompatibility, and sealing capabilities. As these materials will be in direct contact with pulp tissue for long periods, the biocompatibility is of a particular importance. A biocompatible material should not only promote tissue repair, but should also aid or stimulate the reorganization of injured structures [32, 33]. For the determination of biocompatibility of dental materials, a large number of methods have been recommended, with the analysis of cellular reactions* in vitro *being generally considered the initial approach [34].

One of the aims of this study was to evaluate and compare, by the agar disc diffusion test, the antimicrobial activity of different pulp-capping materials: Dycal (Dentsply), Calcicur (Voco), Calcimol LC (Voco), TheraCal LC (Bisco), MTA-Angelus (Angelus), and Biodentine (Septodont). In addition, the cytotoxicity of these pulp-capping cements on rat odontoblast-like MDPC-23 cells was assessed by both MTT and apoptosis assays.

## 2. Materials and Methods

### 2.1. Materials

Six pulp-capping materials were selected for this study: Dycal (Dentsply), Calcicur (Voco), Calcimol LC (Voco), TheraCal LC (Bisco), MTA Angelus (Angelus), and Biodentine (Septodont). Hydrogen peroxide was employed as a control.  Table 1 shows chemical composition of the materials tested: they were prepared in strict compliance with the manufacturers' instructions.

### 2.2. Bacterial Strains and Growth Conditions

The streptococcal strains used in this study were from the Culture Collection of University of Goteborg (CCUG):* Streptococcus mutans* (CCUG 35176),* Streptococcus salivarius* (CCUG 11878), and* Streptococcus sanguis* (CCUG 17826). The cultures were grown and maintained in a Brain Heart Infusion (BHI, Difco, Detroit, MI, USA).* S. mutans* culture medium was supplemented with 10% (v/v) heat-inactivated horse blood serum (Oxoid, Rodano, Milan, Italy) to improve its growth. The culture of all bacterial strains was statically incubated for 16 h at 37°C under aerobic conditions. This overnight culture, used as source for the experiments, was reduced at a final density of 1 × 10^10^ cells/mL as determined by comparing the OD_600_ of the sample with a standard curve relating OD_600_ to cell number.

### 2.3. Antibacterial Test

#### 2.3.1. Agar Disc Diffusion Test

Sterile paper discs (diameter: 6 mm, thickness: 1 mm) from Whatman international, Maidstone, UK, were impregnated with 10 *μ*L of each pulp-capping material. All materials were prepared according to the manufacturers' recommendations. Hydrogen peroxide solution (30% H_2_O_2_) was used as a positive control, whereas the paper disks not impregnated with any material (PD) were considered the negative control. Then, BHI-agar plates were incubated with 1 × 10^7^ cells/mL of an overnight culture of each streptococcal strain at 37°C for 20 minutes. Excess bacterial suspension was removed from the plates and incubated with the paper disks impregnated with the pulp-capping materials at 37°C for 24 h. The diameter of the halo formed around the paper disc (inhibition zone) was measured by the same operator in two perpendicular locations with a millimeter ruler (sliding calliper) with an accuracy of 0.5 mm, after 24 h and 48 h. The size of the inhibition zone was calculated as follows:

(1) size  of  inhibition  zone   =(diameter  of  halo−diameter  of  specimen)  ×12.

All the assays were conducted in triplicate and the results were recorded in terms of the average diameter of the inhibition zone.

### 2.4. Cytotoxicity Assay

#### 2.4.1. Odontoblast Cell Line Culture Condition

The rat odontoblast-like cell line (MDPC-23) was kindly provided by Dr. Jacques Eduardo Nör (Dept. Cariology, Restorative Sciences, Endodontics; University of Michigan School of Dentistry). MDPC-23 cells were cultured in DMEM medium (Biowhittaker, Rome, Italy) supplemented with 10% fetal bovine serum (FBS), 2% glutamine, 2% sodium pyruvate, 1% amphotericin, and 1% (w/v) streptomycin/penicillin at 37°C in 5% CO_2_ atmosphere [35]. The cells were routinely detached using a trypsin-EDTA solution for 2 min at 37°C and resuspended in DMEM medium.

For the cytotoxicity tests, MDPC-23 cells were deposited in the lower chamber of the 24-well culture plate and left for 4 h at 37°C before any experiment.

### 2.5. Cytotoxicity Tests

We performed the cytotoxicity tests with the Transwell insert (Sigma-Aldrich, St. Louis, MO, USA) methodology and the immortalized rat odontoblast cell line MDPC-23. Cytotoxicity of the six pulp-capping materials was assessed with MDPC-23 cells grown in the lower chamber of a 24 mm diameter Transwell plate with a 0.3 mm pore size polycarbonate membrane (Sigma-Aldrich) [36].

Each pulp-capping material was mixed (Dycal (Dentsply), Calcicur (Voco), MTA Angelus (Angelus), and Biodentine (Septodont)) following the manufacturer's instructions onto paper disks or cured (Calcimol LC (Voco), TheraCal LC (Bisco)) by a halogen lamp (Elipar Trilight, 3M-ESPE) for 20 s at 800 mW/cm^2^, and all were placed in the Transwell membrane of the inner chamber.

The Transwell membrane of the inner chamber containing the pulp-capping materials was then placed into the lower chamber of the 24-well culture plate containing at the bottom 5 × 10^4^ cells/well and incubated at 37°C in 5% CO_2_ atmosphere for 24 h, 48 h, and 72 h, respectively. Some wells were incubated with only tissue culture media (negative control), whereas others were incubated with a 10% dilution of 30% H_2_O_2_ (positive control). At the end of each incubation time the cell viability was performed with MTT test. The results were presented as percentage of cell viability with respect to cells incubated in absence of pulp-capping materials set at 100%. The MDPC-23 treated with H_2_O_2_ did not show cell viability (data not shown). Five replicates for each pulp-capping material were used for each experiment performed in duplicate.

### 2.6. 3-(4,5-Dimethylthiazole-2-yl)-2,5-diphenyl Tetrazolium Bromide (MTT) Test

To evaluate the mitochondrial activity of MDPC-23 cells, a test with 3-(4,5-dimethylthiazole-2-yl)-2,5-diphenyl tetrazolium bromide (MTT; Sigma-Aldrich, St. Louis, MO, USA) was performed after 24 h, 48 h, and 72 h as previously reported [37]. Aliquots of 200 *μ*L were sampled, and the related absorbance values were measured at 570 nm by a microplate reader (BioRad Laboratories, Hercules, CA, USA). A standard cell viability curve was used and the results were expressed as a percentage in relation to the untreated cells, respectively.

### 2.7. Apoptosis

An early event in apoptosis is the exposure of phosphatidylserine (PS) residues at the outer plasma membrane leaflet [38]. To determine the exposure of PS, cells were stained with an analog of Annexin V, PSVue480, according to the manufacturer's instructions (Molecular Targeting Technologies, West Chester, PA, USA). PSVue reagents are a family of fluorescent probes containing a bis(zinc^2+^dipicolylamine) group (Zn-DPA), a motif that has been found to bind with high affinity to surfaces enriched with anionic phospholipids, especially phosphatidylserine (PS) exposed on cell membranes. Briefly, MDPC-23 cells were seeded on glass coverslips at a density of 5 × 10^4^ cells per well and incubated with H_2_O_2_ (positive control; 100 mM for 18 h), without pulp-capping materials (negative control), and with each one of the six pulp-capping materials for 48 h, respectively. At the end of each culture condition, cells were stained with PSVue480 solution prepared as follows: a 2 mM solution of preweighed apo-PSS480 was prepared in DMSO until the solid apo-PSS480 was fully dissolved; an equal volume of 4.2 mM zinc nitrate solution was then added; the resulting solution was placed in a water bath at 40°C and shaken frequently for 30 min to ensure complete complexation. A clear orange or red solution of 1 mM stock in 1 : 1 DMSO/water resulted. The samples were stained with 10 *μ*M PSVue480 by gently shaking for 2 h at room temperature and finally washed with *N*-tris(hydroxymethyl) methyl-2-aminoethane sulphonic acid buffer (TES). Then samples were counterstained with a propidium iodide solution (2 *μ*g/mL) to target the cellular nuclei and then observed with a fluorescent microscope at 20x and 40x magnification.

### 2.8. Statistical Analysis

The diameter of the growth inhibition zones was analyzed by analysis of variance (ANOVA). Firstly, data were assessed to be normal by means of Kolmogorov and Smirnov test. The ANOVA and post hoc Tukey test were carried out. Significance was predetermined for *P* < 0.001. Descriptive statistics, including mean, standard deviation, minimum, median, and maximum, were calculated for each group tested.

The distribution of the numbers of vital cells for every pulp-capping material was assessed to be normal with the Kolmogorov and Smirnov test. The data were then analyzed by ANOVA. Post hoc Bonferroni test was applied to investigate the differences among the number of vital cells of the materials. Significance was predetermined for *P* < 0.001.

The analyses were conducted with Stata/SE 12.0 software (College Station, TX, USA).

## 3. Results

### 3.1. Antimicrobial Activity

**Figure 1:**
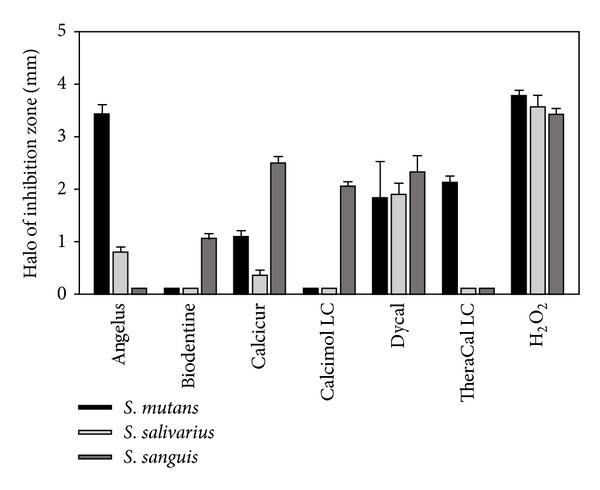
Antibacterial activity of the different pulp-capping materials evaluated by agar diffusion test. Each paper disk impregnated with the different pulp-capping materials was placed on agar plates previously incubated with the indicated streptococcal strains and incubated at 37°C for 24 h. The positive control was represented by a 10% dilution of 30% H_2_O_2_. All the assays were conducted in triplicate and the results were recorded in terms of the average diameter of inhibition zone (mm). Error bars indicate standard errors of the means.

The antimicrobial activity of the tested pulp-capping materials was evaluated with the agar disk diffusion test (Figure 1). As shown in Figure 1, the results were quite different among the three streptococcal strains and the pulp-capping materials. MTA-Angelus (Angelus), TheraCal LC (Bisco), Dycal (Dentsply), and Calcicur (Voco) showed a decreasing antibacterial effect on* S. mutans*; only Dycal (Dentsply) was effective against* S. salivarius*; Calcicur (Voco), Dycal (Dentsply), and Calcimol LC (Voco), followed by Biodentine (Septodont), were effective on* S. sanguis*. Dycal (Dentsply) was the only pulp-capping material showing a discreet antibacterial effect against all the three streptococcal strains.

For the investigation of the antibacterial properties, the ANOVA showed the presence of significant differences among the various groups. Tukey test showed that when testing antibacterial activity with* Streptococcus salivarius*, the highest growth inhibition values (*P* < 0.001) were reported with Dycal (Dentsply). MTA-Angelus (Angelus) showed significantly lower values than Dycal (Dentsply) and significantly higher values than all other pulp-capping materials (*P* < 0.001). Calcimol LC (Voco) and TheraCal LC (Bisco) showed no significant differences between them (*P* > 0.05) and all showed significantly lower values than Biodentine (Septodont) and Calcicur (Voco).

When testing antibacterial activity with* Streptococcus sanguis*, the highest growth inhibition values (*P* < 0.001) were reported with Dycal (Dentsply) and Calcicur (Voco). The lowest growth inhibition values (*P* < 0.001) were reported with MTA-Angelus (Angelus) and TheraCal LC (Bisco). Biodentine (Septodont) and Calcimol LC (Voco) showed significantly lower values than Dycal (Dentsply) and Calcicur (Voco) and significantly higher values than all other adhesives tested (*P* < 0.05).

When testing antibacterial activity with* Streptococcus mutans,* the highest growth inhibition values were reported with MTA-Angelus (Angelus) (*P* < 0.001). Significantly lower values were reported with TheraCal LC (Bisco) and Dycal (Dentsply) that showed significantly higher values than Biodentine (Septodont), Calcimol LC (Voco), and Calcicur (Voco) (*P* < 0.05).

### 3.2. Cytocompatibility Study with MDPC-23 Cells

**Figure 2:**
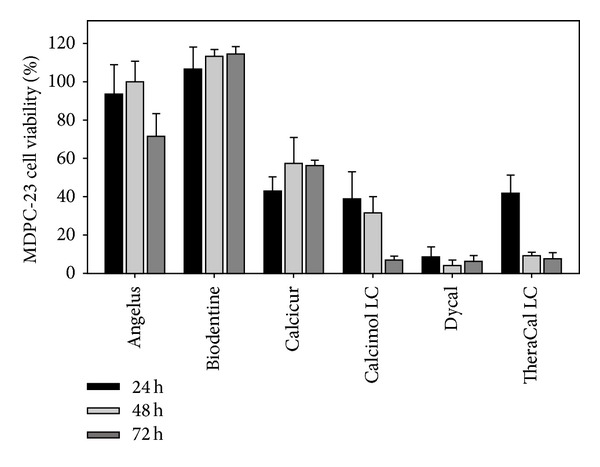
MDPC-23 cells cytocompatibility of the different pulp-capping materials using the Transwell method. MDPC-23 cells were incubated with the different pulp-capping materials at 37°C for 24 h, 48 h, and 72 h in a Transwell culture plate as reported in Materials and Methods Section. The cell viability was assessed with MMT test. The data are presented as percent of the control incubated in absence of any materials and set as 100%. Five replicates for each pulp-capping material were used for each experiment performed in duplicate. Error bars indicate standard errors of the means.

The cytocompatibility investigations were performed with all materials at different times of culture with MDPC-23 cells (Figure 2). As shown in Figure 2, Biodentine (Septodont) and MTA-Angelus (Angelus) showed the highest percent of cell cytocompatibility if compared to the other pulp-capping materials. Biodentine (Septodont) did not show any difference in cell viability at the three incubation times, whereas MTA-Angelus (Angelus) shows a lower cytocompatibility at 72 h (70%); Calcicur (Voco) showed a discrete cell cytocompatibility (50–60%) whereas Calcimol LC (Voco) and TheraCal LC (Bisco) show a very low cytocompatibility but only at longer incubation time (72 h). Dycal (Dentsply) showed the lowest cytocompatibility (10% cell viability) among the pulp-capping materials independently of the culture times.

At 24 h, the highest numbers of viable cells were obtained with Biodentine (Septodont) and MTA-Angelus (Angelus) (*P* < 0.001). Calcimol LC (Voco), TheraCal LC (Bisco), and Calcicur (Voco) presented cytocompatibility values significantly lower than Biodentine (Septodont) and MTA-Angelus (Angelus). Dycal (Dentsply) showed the lowest cytocompatibility (*P* < 0.001).

After 48 h, MTA-Angelus (Angelus) and Biodentine (Septodont) showed no significant differences in cytocompatibility (*P* < 0.001). The lowest cytocompatibility was shown by Dycal (Dentsply) and TheraCal LC (Bisco) (*P* < 0.001), while Calcicur (Voco) and Calcimol LC (Voco) presented a cytocompatibility better than Dycal (Dentsply) and TheraCal LC (Bisco) (*P* < 0.001).

After 72 h, the highest cytocompatibility was obtained with Biodentine (Septodont) (*P* < 0.001). MTA-Angelus (Angelus) showed a little lower cytocompatibility (*P* < 0.001). The lowest cytocompatibility was obtained with Dycal (Dentsply), Calcimol LC (Voco), and TheraCal LC (Bisco) (*P* < 0.001) while Calcicur (Voco) showed an intermediate cytocompatibility (*P* < 0.001).

**Figure 3:**
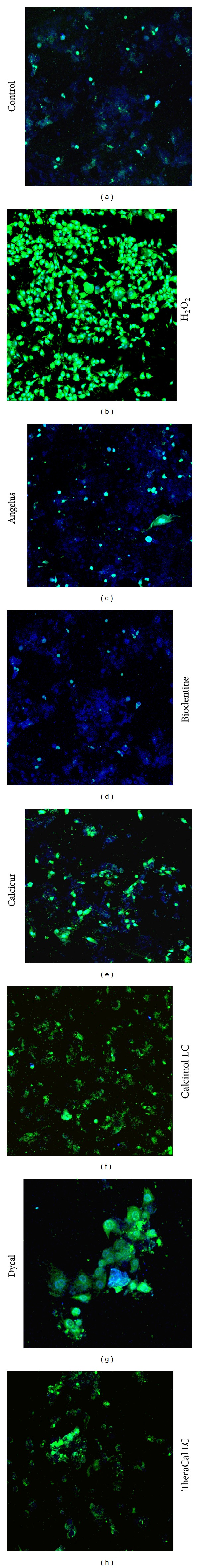
CLSM images of apoptosis assay. MDPC-23 cells were cultured in a 24-Transwell-tissue culture plate for 24 h at 37°C in the absence of any material (a) or in the presence of H_2_O_2_ (b), MTA Angelus (c), Biodentine (d), Calcicur (e), Calcimol LC (f), Dycal (g), and TheraCal LC (h). PSVue480 reagent was used for staining apoptotic cells. CLSM images were taken at 20x magnification.

In Figure 3, the CLSM images representative of MDPC-23 cells indirectly cultivated with the different pulp-capping materials for 24 h and stained with PSVue480 reagent to evaluate cell apoptosis are reported. In absence of any type of materials, the cells were not green fluorescent (Figure 3(a)) and the nuclei turned stained with Hoechst, which usually stains live cells. H_2_O_2_ is very cytotoxic and the cells stained with PSVue480 reagent are completely fluorescent in green (Figure 3(b)). The CLSM images obtained after incubation with different pulp-capping materials confirmed the MTT assay results: MTA-Angelus (Angelus) (Figure 3(c)) and Biodentine (Septodont) (Figure 3(d)) were not cytotoxic whereas Calcicur (Voco) (Figure 3(e)) showed some cells fluorescent in green; Calcimol LC (Voco) (Figure 3(f)) and TheraCal (Bisco) (Figure 3(h)) were very slightly cytotoxic if compared to the negative control (Figure 3(a)); a few cells were observed in the presence of Dycal (Dentsply), indicating a high level of cell cytotoxicity (Figure 3(g)).

At 24 h, the highest numbers of viable cells were obtained with Biodentine (Septodont) and MTA-Angelus (Angelus) (*P* < 0.001). Calcimol LC (Voco), TheraCal LC (Bisco), and Calcicur (Voco) presented cytocompatibility values significantly lower than Biodentine (Septodont) and MTA-Angelus (Angelus). Dycal (Dentsply) showed the lowest cytocompatibility (*P* < 0.001).

After 48 h, MTA-Angelus (Angelus) and Biodentine (Septodont) showed no significant differences in cytocompatibility (*P* < 0.001). The lowest cytocompatibility was shown by Dycal (Dentsply) and TheraCal LC (Bisco) (*P* < 0.001), while Calcicur (Voco) and Calcimol LC (Voco) presented a cytocompatibility better than Dycal (Dentsply) and TheraCal LC (Bisco) (*P* < 0.001).

After 72 h, the highest cytocompatibility was obtained with Biodentine (Septodont) (*P* < 0.001). MTA-Angelus (Angelus) showed lower cytocompatibility (*P* < 0.001). The lowest cytocompatibility was obtained with Dycal (Dentsply), Calcimol LC (Voco), and TheraCal LC (Bisco) (*P* < 0.001), while Calcicur (Voco) showed an intermediate cytocompatibility (*P* < 0.001).

## 4. Discussion 

Microorganisms are considered the primary etiological agents in endodontic disease [39]. The agar diffusion test has been widely used to evaluate the antibacterial activity of dental materials [39–41]. The advantage of the agar diffusion test is that it allows direct comparisons of materials against the tested microorganisms, while a great disadvantage of this method is that it does not distinguish between microbiostatic and microbicidal properties of the materials [42]. Several factors that are relevant to the diffusion capacity of materials in agar must be considered, such as the contact between the experimental material and agar, molecular weight, size and shape of the antimicrobial agent, load and concentration of the test material, agar gel viscosity, and ionic concentration in relation to the medium. Furthermore, the control and standardization of the inoculation density, evaluation of results, selection of agar medium, incubation temperature of plates, and reading point of inhibition haloes are restricting factors affecting the dynamics and variability of diffusion tests in an agar medium [43].

Nevertheless, if most of these variables are carefully controlled, consistent and reproducible results may be obtained. Because of the obvious limitations of* in vitro *studies, clinical inferences should be drawn with strict caution. In our study, we obtained* in vitro* results that underline the antibacterial effects mainly for calcium hydroxide-based materials, as Dycal (Dentsply) and Calcicur (Voco). Similar results were obtained for Calcimol LC (Voco) and TheraCal LC (Bisco), both of which are light-curing materials, although the sensibility of the halo of inhibition zone was different among the species of microorganisms. MTA-based materials such as MTA-Angelus (Angelus) and Biodentine (Septodont) showed a variable effect against the different streptococci.

In the first step of our study on the antimicrobial effects of different pulp-capping materials, we confirmed that calcium hydroxide has an antibacterial activity, as reported in previous studies [44]. The antibacterial activity of Ca(OH)_2_ is related to the release of hydroxyl ions in an aqueous environment [45]. Hydroxyl ions are highly oxidant free radicals that show extreme reactivity with several biomolecules. This reactivity is high and indiscriminate, so this free radical rarely diffuses away from its site of generation [36]. However, this indiscriminate action also affects the mitochondrial activity of cultured cells when testing the cytocompatibility.

The antimicrobial effects of MTA-based materials are not yet well known. MTA consists of 50% to 75% (by weight) of calcium oxide and 15% to 25% of silicon dioxide. These two components together comprise 70% to 95% of the cement. When these raw materials are blended, they produce tricalcium silicate, dicalcium silicate, tricalcium aluminate, and tetracalcium aluminoferrite. When water is added, the cement hydrates to form silicate hydrate gel. It has been shown that, on hydration, MTA produces calcium hydroxide. Thus, it can be concluded that both MTA and calcium hydroxide may have a similar mechanism of action against bacteria [46].

Many studies have evaluated the effect of MTA on microorganisms, but with conflicting results [47–49]. Ribeiro et al. [50] suggested that these variations might be the results of the methodology used, such as aerobic and anaerobic incubations. It has been shown that in an aerobic atmosphere MTA can generate reactive oxygen species which, as reported above, have an antimicrobial activity similar to that obtained with calcium hydroxide. However, under anaerobic conditions, a decrease in the generation of radicals was observed [51]. Ribeiro et al. [50] reported that, in an anaerobic atmosphere, MTA was incapable of generating the radicals responsible for the antimicrobial effect on the different bacterial strains. In addition, Torabinejad et al. found that MTA had no antibacterial effect against any of the strict anaerobic bacteria [38]. However, as shown by our results, it is possible that MTA highly alkaline pH of 12.5 affords its antimicrobial activity [52] even when it acts in anaerobic conditions.

Exposing pulp tissue can cause inflammation and necrosis of the pulp itself, forcing the clinician to use an endodontic treatment. Pulp-capping materials should act as a barrier protecting the vitality of the entire pulp tissue by covering the minimal exposed tissue and by preventing the need for further endodontic treatments. Consequently, the material used should provide an appropriate host response. This means that tissues that are in contact with the materials must not present any toxic, irritating, inflammatory, allergic, genotoxic, or carcinogenic action [53]. In this study, we chose to use the Transwell insert methodology, which is a nondirect contact test [36] for the biocompatibility study. The advantage of using a nondirect contact test for the evaluation of the dental material cytotoxicity is related to the fact that cells and materials are usually separated. Furthermore, various different* in vitro* barrier tests have been already developed [54–57].

Our results indicate some considerable negative effects after the application of each of the materials tested on the culture plate, with the exception of Biodentine (Septodont). As shown in Figure 2, the decrease in the number of cells in the culture plate is sizeable for calcium hydroxide-based materials. Nonetheless, calcium hydroxide solutions have been largely used because of their property of stimulating dentin formation. In clinical practice, the presence of hard tissue barrier after capping can be considered an asset, since it provides natural protection against the infiltration of bacteria and chemical products [58]. However, the importance of calcified hard tissue barrier formation after capping has been challenged by other studies, which have shown multiple tunnel defects and cell inclusions in bridges following pulp-capping with calcium hydroxide [59, 60]. This may lead to leakage and bacterial penetration into pulp tissue unlike the permanent seal produced by bonding agents. For both of these reasons, calcium hydroxide does not seem an eligible material to be used in the case of exposed pulp tissue [61]. In this study, the* in vitro* cytocompatibility analysis showed a better percentage of cell viability for MTA-based materials such as Biodentine (Septodont) and MTA-Angelus (Angelus), although an initial cytotoxic effect was recorded, which may be attributable to the high pH value of the composition of each material. During the 72 h of application of Biodentine (Septodont) on the culture plate, the modifications that occurred underlined the positive trend of the mitochondrial activity, although the differences between 24 h and 72 h were not statistically significant. The remnant calcium hydroxide-based materials showed a reduction in the percentage of cell viability after 72 h, suggesting a different rate of cytocompatibility over the long-term period. In accordance with Hwang et al. [62], the present study emphasized the fact that MTA-based pulp-capping materials do not present cytotoxicity when tested by MTT assay. Other researchers using a short-term* in vivo* assay [63], capable of generating well-founded clinical inferences, also confirm our* in vitro* results. In the last decade, many experimental and clinical studies have been carried out to develop and test new materials and new procedures endowed with safe biocompatibility and anti-infective properties [64–68].

## 5. Conclusions

MTA-based products show lower cytotoxicity and valuable antibacterial activity, unlike calcium hydroxide-based materials, which present not only higher antibacterial activity but also higher cytotoxicity. However, the conclusion that MTA-based pulp-capping material does not show cytotoxic effects* in vitro* should be taken with caution because the experimental design* in vitro *has some inevitable limitations with respect to the* in vivo* situation, where cellular responses and inflammatory and/or reparative reactions may differently influence the effects.

## Figures and Tables

**Table 1:** Characteristics of tested materials.

Material	Components	pH	Manufacturer
Dycal	Two-paste system made of a base paste (1,3-butylene glycol disalicylate, zinc oxide, calcium phosphate, calcium tungstate, and iron oxide pigments) and a catalyst paste (calcium hydroxide, N-ethyl-o/p-toluene sulphonamide, zinc oxide, titanium oxide, zinc stearate, and iron oxide pigments)	9–11	Dentsply Tulsa Dental, Johnson City, TN, USA

Calcicur	Water-based calcium dihydroxide paste	12.5	Voco GmbH, Cuxhaven, Germany

Calcimol LC	Light-curing radiopaque one-component material containing urethane dimethacrylate resin, calcium dihydroxide, dimethylaminoethyl-methacrylate, and TEGDMA	10–12	Voco GmbH, Cuxhaven, Germany

TheraCal LC	Light-curing, resin-modified calcium silicate filled liner single paste containing CaO, calcium silicate particles (type III Portland cement), Sr glass, fumed silica, barium sulphate, barium zirconate, and resin containing Bis-GMA and PEGDMA	10-11	Bisco Inc., Schamburg, IL, USA

MTA-Angelus	Powder containing type III Portland cement, bismuth oxide, tricalcium silicate, dicalcium silicate, and tricalcium aluminate tetracalcium aluminoferrite	12	Angelus, Londrina, PR, Brazil

Biodentine	Powder containing tricalcium silicate, calcium carbonate, and zirconium oxide. Liquid containing water, calcium chloride (accelerator), and modified polycarboxylate	12	Septodont, Saint-Maur-des-Fosses, France
